# Annotated bibliography on participatory consultations to help aid the inclusion of marginalized perspectives in setting policy agendas

**DOI:** 10.1186/s12939-014-0124-0

**Published:** 2014-12-20

**Authors:** Faraz Rahim Siddiqui

**Affiliations:** O’Neill Institute for National and Global Health Law, Georgetown University Law Center, 600 New Jersey Avenue, NW Washington, DC 20001 USA

**Keywords:** Participation, Consultations, Inclusion, Marginalization, Post-2015, MDGs, Priorities, Frameworks, Methods

## Abstract

The purpose of this bibliography is to present studies from peer-reviewed and grey literature that used consultations and other participatory strategies to capture a community’s perspective of their health priorities, and of techniques used to elevate participation from the implementation phase to a more upstream phase of prioritization, policymaking and agenda setting. The focus here is of those studies that worked with marginalized populations or sub-populations. This bibliography contains four areas of research. It begins by first offering some philosophical and conceptual frameworks that link participatory interventions with inclusive policy making or agenda setting, and a rationale for prioritizing marginalized populations in such an undertaking. After situating ourselves in this manner, the second section looks at various participatory instruments for participatory consultations, for reaching out to marginalized populations, and for communicating the results to policymakers. Two sets of distinctions are made here: one between external (non-invitation) and internal (stifling of opinions) exclusion, and between mere participation and from active inclusion within consultations and within the policies. In the third section, examples of consultations that created or changed policy in various jurisdictions are shared, followed by a final section on a reflective and evaluative look at the recruitment, instruments and examples. An earlier iteration of this bibliography was created to assist a multi-country research project by the author to inform the UN Post-2015 development framework of the views of several diverse and highly marginalized populations around the world on their health-related priorities.

## Introduction

Health priorities and agendas have been expressed in many forms and have been compiled at several forums through various national and international processes. The Millennium Declaration, which inspired the Millennium Development Goals (MDGs), emphasized the importance of a “more inclusive political process” that allows “a genuine participation by all citizens in all our countries” [1]. Yet, participatory approaches that get information on health needs and priorities by consulting the community—the subject of the health development agendas and the users of health services and systems—are not common. In fact, traditional priority-setting initiatives have preferred technical approaches such as the burden of disease information and cost-effectiveness considerations to set priorities [2].

Several disadvantages with these common methods are becoming increasingly clear in the global development community. First, the utilitarian nature of the technical approaches fails to account for a wide range of values that are involved in making priority-setting choices [1] and may miss priorities that better reflect the community’s needs and experience. Second, without community consultations, these technical approaches have largely been driven by supply-side issues such as funding availability and donor interests, contributing to creating global health policy and programs that are vertical and directed at select diseases instead of those that promote horizontal, system-wide integration and address the underlying determinants of health [3]. Letting the community share their needs and challenges provides a more holistic, complex and interrelated experience. Finally, using strategies like efficiency and cost-effectiveness alone can exclude the concerns of marginalized groups. Implementation tends to be both more expensive, given that they often have the most complex barriers, and less cost-effective, because of the lack of the economies of scale [4,5]. This is despite the fact that marginalized groups are often most disproportionately affected by poor health.

With the approaching deadline of achieving the Millennium Development Goals (MDGs) and the development of the Post-2015 New Development Goals, participatory strategy that incorporate the needs and priorities of marginalized groups are been explored and implemented [6,7]. Go4Health is a global consortium of academics and members of civil society tasked with advising the European Commission on new global health-related goals to follow the MDGs. To increase the legitimacy and transparency of this advice, Go4Health is committed to including the voice of communities and marginalized populations around the world in this process so that the goals proposed are more equitable and relevant to people who are generally excluded from global processes. A participatory method has several advantages over other non-participatory methods. The goals thus produced will be inherently more credible, genuinely attuned to the understanding of the users of the health system as opposed to goals that are based on the perceptions and assumptions of policymakers (who are frequently from a different socioeconomic status, with different life experiences) and are applied under the pretext of economic feasibility [8,9]. Moreover, an inclusive process that meaningfully engages communities can help gain community trust and buy-in to adopting these goals and working with the health systems towards achieving them [9]. There is also evidence on the role of participatory approaches in reducing conflict, and helping with constituent support [10]. Finally, if consultations engage marginalized populations, they will be more likely to address the greatest barriers to health equity in their community. Marginalized communities may reveal their experience with barriers to good health that extend beyond the health sector, possibly addressing issues related to socioeconomic struggles, stigma, transport, and more. Differences in the needs of these sub-populations may emerge to reframe health priorities [11].

To this end, one of the Go4Health working groups comprised of partners from academic institutions and community-based organizations has been assigned to undertake dialogues with communities and civil society to gain an understanding of their priority health needs. These researchers are geographically diverse, with regional hubs based in Africa, South America, Asia and Australia, and target communities based in rural or urban settings in up to three countries per region. Regional hubs are led by universities or civil-society based organizations with expertise in community-based work and strong research experience in their respective regions.

## Methods and limitations

This bibliography was compiled in December 2013 through a two-stage process. For the first stage, a purposive sample was generated by snowball sampling by Go4Health partners around participatory research methods. Purposive sampling, a non-randomized method, was an appropriate place to begin the research for this paper since it relies on expert knowledge on a subject and on the characteristics that are important to be represented in the sample [12]. It was also suitable for the unique task of contributing to a global development agenda using community input from multiple countries by researchers from several academic and civil-society based organizations. This initial list of 12 studies was studied in depth.

The second stage consisted of a search of journal databases. The databases used were PubMed (73), Scopus (215, plus 250 on web), Web of Knowledge (95), Cochrane Method Studies (23) and Equinet (24; equinetafrica.org) and resulted in 417 unique articles. This stage broadened the evidence, issues and examples for this project. The search was conducted using several combinations of key terms reflecting the criteria described below.

These criteria define the dimensions on which the studies were assessed for inclusion and analysis. They are loosely ranked in terms of their importance to this bibliography. As such, the final choice of articles listed includes some that may not fully address the third or fourth criteria (cases when this occurred are listed as additional inclusion criteria below). This was done in an attempt to provide an adequate treatment of each criterion rather than rely on a smaller sample of studies that address all the criteria at once. However, the bibliography as a whole addresses each criterion sufficiently, and several studies create associations between all the criteria.

1. **Community as a stakeholder:** studies that enhance the understanding of why the community (“civil society”; “public at large”) is an important stakeholder and why their values and perspective as subjects of the policies or services being developed should be sought. *Additional inclusion criteria*: studies that gain the community’s perspective using “proxy” measures (i.e., without fulfilling criterion #2) are presented in the last section as possible methods to triangulate or validate the community data, not as alternatives for “meaningful engagement.”
2. **Meaningful engagement of community:** input on community needs and demands is collected through direct consultative and participatory methods, including input on the public’s role and rights on the engagement process itself, and on the development and implementation process in general. *Additional inclusion criteria*: study methods enhancing the community’s understanding of their own needs and rights.
3. **Marginalized populations:** The research should include meaningful participation from groups that represent marginalized or highly vulnerable populations that are systemically excluded from national or international policy making forums (for example: refugees, indigenous, lowest caste). The studies employ credible and valid methods to recruit and consult these populations.
4. **Determining priorities:** Studies that helped determine policy, research or development agendas or priorities were given precedence, since they involved the community in the early phase of identifying and assessing important needs. This is an important goal of our project and that of the Post-2015 UN consultations. *Additional inclusion criteria*: studies involving rationing or prioritizing amongst pre-determined needs (under experimental or budget constraints, for example) are also included to review methods on deliberating between multiple needs, which may be useful.

The article titles and abstracts were analyzed for relevancy to the topic and how well they matched the criteria, resulting in a shorter list of 76 articles that adequately addressed all the elements important to the current bibliography. These papers were then analyzed according to a framework of research questions developed from an analysis of the articles in stage 1.

The final list consists of a representative selection of reviews, research articles, interventions, services and case studies from the sample of peer-reviewed journals, conferences, and government or NGO reports. Five research questions were used to arrange the bibliography into sub-headings, and the articles within each sub-heading were ordered according to how well they responded to the relevant questions.

For search terms, database specific codes were developed for the keywords below, in decreasing order of weight:

1. participated OR Participatory OR participation OR included OR inclusive OR inclusion OR engaged OR engagement OR involved OR involvement **AND**
2. Marginalization OR marginalized OR marginalisation OR marginalised OR vulnerable OR vulnerability OR excluded OR exclusion OR segregated OR segregation OR discriminated OR discrimination OR disadvantaged OR minority **AND**
3. Consultation OR consulted OR dialogue OR dialog OR meeting OR conference OR assembly **AND**
4. Views OR opinions OR suggestions OR perspectives OR assessments OR demands OR entitlements OR preferences OR desires **AND**
5. civil society OR local OR Public OR Citizen OR Community OR population OR group **AND**
6. Priority OR priorities OR needs OR rationing OR goals OR agenda.

Important exclusion terms were: individual “needs”; research “priorities” or “goals”; “participation” in clinical trial; setting organization or university “agenda”; medical “vulnerability”; medical “consultation”. Exclusion criteria were: studies that observed practices instead of consulting on needs; when the policy did not affect the participants; and when the vulnerability of the participants was a result and was not known going in.

There were two major limitations in this paper. Although some non-English sources were extensively studied, most of the research presented was written in English. In the same way, the sources from stage 1 were mostly from grey literature, but only a handful of the sources listed below are not journal articles. Finally, the research was driven by its relevancy to Go4Health’s project to gather evidence for post-2015 priorities from communities, which could have biased the final selection of resources.

This document is fairly comprehensive in the issues it covers but does not provide a systematic synthesis of the papers presented in the annotations or the section introductions, either of the themes discussed or of the content analysed.

## Bibliography with annotations

A collection of previous work and research on participatory consultations with community on their health priorities can help develop a conceptual framework and select the best methods and techniques for participatory community consultations. The sub-headings below represent key themes in the literature that respond to the analysis framework based on the following key questions:

1. *What values and frameworks have researchers appealed to for including community participation, specifically by marginalized groups?*
2. *What are the participatory approaches that have been used to consult a community on their needs and health priorities? What are credible strategies consulting marginalized populations? What are some best practices in taking their voices to policymakers?*
3. *What are the best practices for achieving rigorous, credible participation with marginalized populations within a community?*
4. *What were some conceptual and logistical challenges with including the views of the community in each of the stages of creating participatory consultations?*

### Underlying values and frameworks for participatory priority research

There are a wide variety of participation strategies to include a community in the implementation of health research or interventions (for example, see [13,14]). However, research shows that community input can often be insufficient, such as when it ends too early [15,16] or when it addresses too few dimensions [17]. To avoid such issues of comprehensiveness and systematic application, the first step towards participatory agenda setting is to use a framework that ties together the underlying objectives, values and policy imperatives to the participatory priority research methods and formats. This section shares some such frameworks from the literature.

Byskov J, Bloch P, Blystad A, Hurtig A-K, Fylkesnes K, Kamuzora P: **Accountable priority setting for trust in health systems - the need for research into a new approach for strengthening sustainable health action in developing countries.***Health Research Policy and Systems* 2009, **7**:23. To formulate a thoroughly participatory agenda-setting or policy development system, Byskov et al. (2009) start on a normative plane: they appeal to an ethical framework called Accountability for Reasonableness (A4R) that seeks “legitimacy and fairness” in priority setting. The original conceptualization of the A4R framework by Daniels and Sabin accepted that people may “justifiably disagree” on the relevant values to consider when making priorities, but that all values “centre on fairness, on which there will be no disagreement” [18]. In doing so, Byskov et al. give participatory policy making a philosophical foundation. They then allow for *and* present a framework to select a number of community-defined values on which health interventions should be based. The authors bring participation in the plane of values, this is far from an abstract exercise: they refer to instances and forums in which they have worked with local populations to develop the values on which they would like to build their health systems and services. They furnish an example of A4R framework implementation at a district level in Tanzania. Here, it helped in strengthening transparency, accountability, stakeholder engagement, and fairness.

Paul, S: **Community Participation in Development Projects: The World Bank Experience.***World Bank Discussion Papers* 1987. Washington, DC: World Bank, pp. 2–11. A conceptual framework for community participation in general was proposed by the World Bank in 1987. Community participation was conceptualized over three axes and on their relationships: the objectives of participation (included any of empowerment, capacity building, effectiveness, cost sharing and efficiency); its intensity (from low to high intensity, these would be information sharing, consultation, decision making and action initiation); and its instruments (participation by user groups, field staff or community panels). The World Bank’s community participation had an explicit emphasis on equity and benefit sharing by the poor. The authors noted that conceptualization at this level would have several implications for the Bank’s developmental policies. Firstly, the objectives favoured by policymakers (efficiency) and researchers (effectiveness) are set up against those most important to the community (capacity building), aiding participation from the earliest stages of creating the development agenda. In addition, the proposed framework explicitly emphasized the centrality of empowering the poor to allow them to provide feedback on proposed interventions and create more equitable policies. This theme carries into the other axes, calling for negotiations between the project authorities and the beneficiaries in intensity and instruments. Most importantly, the framework is just that: an overall model which, using the principles of participation, allows for customized strategies for each situation.

*See also:* Charles C and DeMaio S: **Lay participation in health care decision making: a conceptual framework**. *Journal of Health Politics, Policy and Law* 1993, 18(4):881–904. This paper provides another conceptual framework of community participation which looks at the degree of participation, domains where decision-making is needed and the different roles that need to be included. This is another framework that can allow a development program to holistically consider its objectives and methods so as to help motivate “lay participation” and develop participatory decision making.

Habib, A: **South Africa: Conceptualising a politics of human-oriented development**, 2008. Social Dynamics, 34(1), 46–61. This paper from South Africa offers a political model of development which, if sufficiently convincing, can open up the literature and traditions of political participation to serve as the rationale incentives for participation in development agenda setting. Habib (2008) challenges the notion that participatory development and representative democracy are distinct systems and to achieve the former proposes several political changes. He notes that the strategies and policies he recommends, such as the emergence of an independent, robust, plural civil society, suggest not only that human‐ oriented development is a product of a political process, but also that it requires an intricate mix of representative and participatory democratic elements.” His strategies simultaneously strengthen the participatory character of the political system and provide a voice to the poor. In conclusion, he uses the last two decades of the South African experience to make a strong argument that the political environment around development needs to be interrogated in an inclusive manner to create sustainable policy shifts towards greater societal participation, greater accountability between the political elites and citizens and respect for the interests of the poor and marginalized.

*See also:* Palmer, L: **“Nature”, place and the recognition of Indigenous polities**, 2006. Australian Geographer, 37(1), 33–43. In a similar line of argument as Habib (2008), the author argues that the “politicization” of nature can allow indigenous populations greater participation in land and resource management initiatives by their governments.

London L: **Issues of equity are also issues of rights: Lessons from experiences in Southern Africa.***BMC Public Health* 2007, 7. Using the underlying premise that equity is good for public health, the author conducted archival reviews and stakeholder interviews of three case studies in Southern Africa to explore how a human rights approach to healthcare can promote health equity. The results illustrated that two factors were critical for a rights-based approach to promote equity. Firstly, the full range of rights would have to be considered, from civil and political rights to the socio-economic and group-based rights. Secondly, the rights-based approach has to be coupled with community engagement in ways that reinforce community capacity, especially when it prioritizes and affords agency to the most vulnerable groups in the society. The conclusion provided a framework with health equity as a goal, and community engagement of vulnerable groups and individuals as the method to achieve that goal.

Reed BJ and Coates S: **Engineering and gender issues-evidence from low-income countries. Proceedings of the Institution of Civil Engineers.***Municipal Engineer* 2003, **156**(2), 127–133. The central assertion of this paper is this: given that engineers can have a great impact on society by developing the tools and infrastructure to reduce the burden of everyday chores (such as getting fuel) or environmental hazards (for example, poor sanitation), if they focus on addressing the problems of the marginalized members of society, they can have a great impact on reducing their burdens, and as such, contribute towards creating a more equitable development process. The authors used a literature review and semi-structured interviews to study the role of engineers in development, both what it is like and what it should be like, and conduct workshops to teach about how to prioritize the issues of traditionally excluded groups, such as women, for engineers in development. The most important conclusions of the research carried out was that the inclusion of the community in the planning phase of development projects makes them more effective, but involving the most marginalized segments of the populations may address the society’s greatest needs—and produce solutions that everyone can benefit from. However, the authors caution not to let the focus on the marginalized exclude the majority.

#### Discussion

Between them, the articles in this section provide the philosophical and conceptual connection linking participatory interventions with participatory policy making, agenda setting or prioritization exercises. This is done in three levels. Firstly, the philosophical foundation is suggested as incorporating fairness as the minimal requirement. Fairness necessitates values, objectives, formats and instruments to allow fair and equitable participation by all citizens. This is followed by two frameworks that cloth these abstract ideas into a conceptual infrastructure. They are meant to present a comprehensive list of objectives, a wide variety of stakeholders, and a broad array of tools and a plan to integrate them that ensures that interventions are just one part of a holistically participatory attitude that spans all levels of development. The next two papers add the context to development. Far from acting in a vacuum, development is fully a function of the social, economic and political environment in which it exists. Habib’s (2008) assertions demonstrate how only a fundamentally participatory political structure with an engaged and empowered civil society can make space for participatory policy-making. This brings to the fore ingredients such as political will, electoral policies and foreign policy designed to create a “human-oriented development trajectory”. In each one of these papers, the poor, disenfranchised citizen gets a central role as empowered citizen and beneficiary of the development agenda. In this way, it relates back to “fairness” instead of pure equality (which would reinforce existing power differentials).

The final two papers help build the rationale for the remaining element of the goal of this paper, namely that of prioritizing marginalized populations. To argue for the inclusion of marginalized populations, London uses the theoretical concept of equity as the foundation of good public health strategies, thus developing the themes discussed in Paul (1987) and Byskov et al. (2009). Although she uses a relatively small sample size, she creates a helpful framework which makes consulting vulnerable populations and individuals the central theme of equitable health policy generation. Reed and Coates (2003), contribute a more technical argument: involving the most marginalized segments of the populations may address the society’s greatest needs—and produce solutions that everyone can benefit from.

Apart from these values and frameworks, various international legal instruments have outlined justifications for consultative national policy making [19-21] by outlining obligations as goals or values that have to be achieved, but leaving out the methodologies and indicators for the interpretation of lawmakers and researchers around the world. Their methods and outcomes have been evaluated by other researchers for how well they achieved certain obligations [22,23].

### From participatory consultations to inclusive policy

After defining the scope of inclusion above and the normative boundaries of what should be the *basis* of inclusion, we move here to the more descriptive specification of what the *nature* of that inclusion should be, and how it should be undertaken. This section first highlights literature on the considerations around conducting participatory consultations. It then reviews a few participatory tools for soliciting the needs and desires of populations, with a particular emphasis on marginalized people (keeping in mind that this is in itself a vast body of literature that can easily be the subject of its own bibliography) and techniques to present the findings to policymakers for setting health policies and agendas. This prepares us for Experiences with consultations for promoting inclusive policies, which presents and discusses some examples of governments that used consultative processes to create their health agendas.

#### Considerations around inclusion

Peterson ND: **Excluding to include: (Non) participation in Mexican natural resource management.***Agriculture and Human Values* 2011, 28(1): 99–107. Using a case study from a natural resource management project in Mexico, the author looks at the manifestation of exclusion in a planning process that was designed to be participatory and included community meetings and debates. However, rather than being a “neutral tool” for planning, participation became the means for taking control, excluding others, and disavowing the stakes of those parties that were most affected by the decisions. The author drew on deliberative democracy to create a typology of exclusions that she observed in the case study. The most important categorization for our purposes was her distinction between two forms of exclusion that hurt legitimacy, external and internal exclusion. External exclusion covers situations or reasons by which an individual or group is not invited to consultations. Internal exclusion, on the other hand, comes about where issues like power dynamics or meeting format create an environment that do not give participants the space to speak their minds. The author concluded that participation is a necessary but not sufficient element of inclusion. Rather, it is better pictured as an easily manipulated tool that needs to be carefully managed.

Williamson AR: **Public meetings as sources of citizen input: Comparing attendees with citizens at large.***The Social Science Journal,* 2013. Although public meetings are the most frequently used method for obtaining citizen input into public decision-making, Williamson hypothesized that they may not be representative of the community at large, or have the views of the community at large. She first characterized the representativeness of the public meetings on a number of factors, including race, Hispanic ethnicity, and low-income status. Interestingly, the racial and ethnic minorities as well as well as low-income people were over-represented when compared to the county at large. She then compared the results from public meetings to establish spending priorities against a randomized telephone survey she conducted in a Florida county to show that the views of the attendees were different from those of the general population. The findings showed differences between the two population sets on a number of categories such as housing assistance and neighbourhood improvement. However, one issue they both agreed on was to fund services for vulnerable populations such as seniors, persons with disabilities and victims of domestic violence. The author concluded that public meetings deserve more attention that they are given for getting feedback on policies as long as some effort is spent to recruit minority representation.

Welbourn A: **A note on the use of disease problem ranking with relation to socio-economic well-being: an example from Sierra Leone.***RRA Notes,* 1992*,* 16:86–87. This short article shares an example of how participatory methods can help avoid internal and external exclusion as defined by Peterson (2011). The author reports on fieldwork conducted with a village community in Sierra Leone using the Rapid Rural Appraisal methods, a thoroughly participatory system of consultation. The standard practice had been to talk only to men that were generally older and better-off. However, when staff worked with older men, younger men, and with women, they realized that the standard practice was “*an entirely inadequate way* of gauging the complexity of a community’s needs.” Women, for example, had different priorities and problems than either group of men. Their non-invitation to consultations fails to identify and address their problems. Next, the author divided the better-off and worse-off women and asked them to rank their problems. The results between the two groups were completely different, showing that combined consultations fail to bring up the problems of a subset of the group. This is internal exclusion since they were present but their voices were still not heard.

Moinpour, CM, Atkinson JO, Thomas SM, Underwood SM, Harvey C, Parzuchowski J, et al.: **Minority recruitment in the prostate cancer prevention trial.***Annals of epidemiology* 2000, **10**(8): S85-S91. Notwithstanding the difference between recruitment for a clinical trial and recruitment for a health policy consultation effort, this article has an important lesson for the purpose of our bibliography when it comes to recruitment within a minority population. The manuscript describes an effort to recruit African American men for a randomized trial, who comprised only 4% of the subjects. Despite several very involved methods used to increase participation, there was barely any increase in the minority enrolment. Several reasons were provided at a debriefing discussion with the outreach staff. Firstly, all sites noted that a longer timeframe was required to make contacts with members of a minority community, establish trust, and make general outreach before proceeding to the point where the issue at hand can be addressed. Local recruiters may shorten the time needed, but the researchers would need to make them equal staff members. Additionally, issues such as distrust of government-supported research can be mitigated and credibility gained if there are separate education seminars about the disease and methods of prevention.

#### Participatory instruments to capture the voices of the marginalized

Chambers R: **The origins and practice of participatory rural appraisal**. *World development* 1994, 22(7): 953–969. This is one of the earlier review describing a set of widely applicable qualitative, participatory methods of research or consultation that fall under the rubric of participatory rural appraisal, or PRA. Chambers describes PRA as experiential methods that enable local people to share, enhance and analyze their knowledge of life and conditions in the process of the consultation, enabling them to plan and to act instead of just responding to external researchers’ knowledge extraction processes. Chambers states that when communities conduct and analyze their own research, they own the process and the information and produce more relevant and actionable results for the community. Participatory methods described include group brainstorming, stories and case studies, participatory mapping, transect walks and analyses of lived experiences. The strength of these methods is twofold: one, the methods are designed to understand the experience of the community at a much more deep-rooted and immersed level than the standard interviews or town halls do, and secondly, the consultation results are meant to create awareness of the needs and goals of the community, not just for the policymakers, but by the community itself so that they are better informed and able to address their own issues.

Ryan M, Scott DA, Reeves C, Bate A, van Teijlingen ER, Russell EM, Napper M, Robb CM: **Eliciting public preferences for healthcare: a systematic review of techniques.***Health Technol Assess.* 2001, **5**(5):5–40.

In this paper, Ryan et al. systematically review and assess the best techniques to elicit public preferences in priority-setting exercises in healthcare, where priorities can then be used to allocate health system resources and solidify policy. They identify quantitative and qualitative methods and assess them based on acceptability, cost, validity, reliability, generalizability and objectivity. The detailed treatment of a large number of quantitative methods was perhaps their most important contribution. These were categorized under ranking, rating and choice-based techniques. Likert and Guttman scales were the cheapest and simplest methods, but they listed other methods such as qualitative discriminant process, the Allocation of Points, the Standard Gamble that consider the strength of preference, or relative weights of the components that make up a choice. These latter methods have a higher validity and reliability but require much more time and money. The authors also described and assessed qualitative methods. These were divided into individual approaches such as one-on-one interviews and the Delphi technique and group approaches including focus groups, citizens’ juries and case studies. The authors concluded that qualitative studies are better suited for to elicit preferences since they have a more nuanced understanding of the social values.

#### Presenting consultation results to policymakers

Harden A, Oakley A, Brunton G and Fletcher A: **Integrating ‘qualitative’ studies and trials in reviews: reflections from reviews about teenage pregnancy, parenthood and social exclusion [abstract].** 2005. Melbourne, Australia: XIII Cochrane Colloquium. This paper tested a method for integrating data gleaned from qualitative studies with other statistical data and can be useful for finalizing policy recommendations. The authors assert that despite the lower credibility given to qualitative research data as evidence, it has several benefits in clinical trials: it helps determine the appropriateness of interventions, explore heterogeneity in effects, and identify promising interventions to test. The authors conduct a high-quality systematic review of qualitative studies about interventions to decrease teenage pregnancies and synthesised the clinical evidence of controlled trials with qualitative data on young people’s perspectives and experiences. They did this using three steps: a meta-analysis of the participant views; qualitative research coding, and a mixed method that evaluated whether the interventions met the needs of young people. Three themes associated with early parenthood emerged from the qualitative studies: dislike of school; poor material circumstances and unhappy childhood; and low expectations for the future. Comparing these to the content of the controlled trials indicated that both early childhood interventions and youth development programmes were appropriate strategies for reducing unintended teenage pregnancies. The authors concluded that their method for including qualitative studies to trials greatly strengthened the evidence base for informing government strategies and public policy.

Lorenz LS, Kolb B: **Involving the public through participatory visual research methods**. *Health Expectations* 2009, 12(3): 262–274. The authors consider presenting policymakers with an understanding of consumer, community, and health system problems and strengths using the increasingly popular visual consultation methods such as photovoice and photo-elicitation, research methods that seeks to understand a community perspective through cameras provided to its members. Specifically, they wished to consider whether this would be a good method of getting the perspective of the most vulnerable sectors such as disabled and low-SES individuals. The authors explore issues from planning and data analysis to the ethical and cultural concerns in photovoice studies conducted in Morocco and USA. They found that visual data identified health system problems and strengths generally omitted from data gathered using other means. Whereas statistical data can tell policymakers that there is a problem that needs to be addressed, the surprise element of visual data encouraged them to pay attention and take action. Their conclusions conveyed the importance of a variety of data types, of participant-generated data, of visual data to get the voice of vulnerable groups to policymakers.

Rideout C, Gil R, Browne R, Calhoon C, Rey M, Gourevitch M, Trinh-Shevrin C: **Using the Delphi and snow card techniques to build consensus among diverse community and academic stakeholders.***Progress in Community Health Partnerships: Research, Education, and Action* 2013, 7(3):331–339. The challenge with inclusionary consultations is generating a consensus form the diverse perspectives and organizational agendas views that are collected. The New York University Health and Hospitals Corporation used community-based participatory research methods to solicit research priorities from members of a community advisory board and from the project’s steering committee. They first collected the data using the Delphi approach, a multi-method, iterative method consisting of a series of surveys that they administered online. Once a list of priorities was created, they used the snow card approach (a technique to merge brainstormed ideas based on similarities between them) to narrow down the lists to two priority areas, namely cardiovascular disease (CVD)/obesity and mental health. The Delphi approach fostered engagement since it required the stakeholders’ input in the decision making process, and the snow card technique allowed them to organize a large number of discrete ideas. The process helped ensure that NYUHHC research and community engagement strategies are congruent with community priorities.

#### Discussion

The articles in this section provided several substantive elements for this bibliography. We see a description and examples of internal and external forms of exclusion. This typology creates for us a distinction between participation—which we can now see as merely the physical presence in consultations—and inclusion, which is the integration of one’s (ideally, everyone’s) perspectives and ideas into the consultations results that affect policy. While some participatory processes might not be inclusive, Williamson’s article (2013) found that sometimes, these methods may actually attract minorities and low-income groups more than other populations. She posits that this may be the case because these forums spoke to minority issues, and because they represent some of the otherwise limited forums for minorities to get their voice heard. Separately, Moinpour et al. (2000) caution us that true inclusivity is a long-term, time consuming process, not just a series of instruments. Instead, states that want truly inclusive policies would need to create a culture of inclusivity. This suggests that consultations for the sake of policy or agenda setting exercises will seem disingenuous if a government does not have a longer-term attitude of inclusion, and the resulting efforts for inclusion may likely fail. Governments that are not aware of these difficulties can get on the field with over-ambitious goals only to find out that they do not even have enough participation to gain legitimacy, and end up filling in the gaps themselves (for example, see Government of Chhattisgarh, 2005 below).

Some instruments for participatory consultations were also discussed above, but a detailed analysis of the same is outside the purview of this bibliography. The articles that are shared are themselves reviews or collections of methods that the authors feel will avoid the forms of exclusions discussed earlier. Ryan et al. (2001) distinguish between quantitative and qualitative instruments and assess them. They conclude that qualitative studies are better suited for social preferences research. Chambers (1994) on the other hand deals only with qualitative instruments, or rather a specific body of qualitative instruments collectively called “participatory rural appraisal” and describes their strengths over other more standard instruments. These articles should be a good point for the reader to start exploring the instruments she may need for inclusive consultations. A brief scan of the literature would show that studies generally used multiple consultation instruments to add greater reliability of their findings. Surveys and questionnaires were popular quantitative method used, while the most common qualitative methods were interviews and focus group discussions. Quantitative instruments were able to reach more people but the latter were able to create more meaningful participation. The added benefit of qualitative methods is that it would be able to select participation from all segments of people, and if enough marginalized populations are selected, will have strong representation from them. Quantitative data will be vulnerable to the “tyranny of the majority” and dilute the input of the marginalized. Any representative sample will, by definition, capture only a few marginalized voices.

Once the consultations are completed, the results need to be presented to policymakers. The three articles dealing with this issue of translating health priority information to those making decisions have some common strands between them. For example, there is an implicit assumption, a reasonable one to be certain, that policymakers do not have time or the desire to read through scientific data and draw policy lessons. The scientific community and researchers need to capture their findings in a palatable and persuasive format for the policymakers. Lorenz and Kolb (2009) talk about the format, while Rideout et al. (2013) and Harden et al. (2005) focus on the substance, specifically making sense of the diverse needs they capture. However, these techniques do not avoid the theoretical problem that translating consultation results into policy imperatives will invariably include some views at the cost of others. In Challenges and reflection on inclusive policy making, we revisit this issue of consolidating the community’s input to generate a clearer policy agenda.

### Experiences with consultations for promoting inclusive policies

This section presents examples of participatory consultations that were used to create an inclusive agenda. There is at least one example from each level of government, from international and multinational policies, to national, state and municipal government policies. At the end of the section, some of the common shortcomings of some of these processes are analysed. Some more specific examples of how civil society activism can create the space to participate in agenda setting [24-26], and others examples evaluating participatory governance structures [27-29], are included in the references but not annotated due to their tangential relevance.

Gulaid LA, Kiragu K: **Lessons learnt from promising practices in community engagement for the elimination of new HIV infections in children by 2015 and keeping their mothers alive: summary of a desk review.***Journal of the International Aids Society* 2012, **15**(2):17390. This paper presented a review of promising practices in community engagement practices around the world that were part of *The Global Plan Towards the Elimination of New HIV Infections Among Children by 2015 and Keeping their Mothers Alive*. It summarized the promising practices in community engagement that helped achieve these goals. The goal was to look for effective practices that were replicable, sustainable and scalable. In this way, it provides this bibliography with the lessons from multiple first case studies that, on an international level, helped create effective global disease prevention policies. The review was itself participatory, supplementing a literature review with key informant interviews. Several of the promising practices validate and strengthen the lessons we learn in section 2 above, including supporting community activism and capacity, as well as promoting local solutions for decision-making and communications needs. The overall message was that real change requires sustained engagement and the input of stakeholders from small informal groups at the grassroots level right up to the global coalition that makes global policies.

United Nations Development Programme: *Indigenous voices in Asia Pacific: Identifying the Information and Communication Needs of Indigenous Peoples*. Bangkok; 2013. This report summarizes and analyses participatory research undertaken between 2007 and 2011 in Cambodia, Indonesia, Lao PDR, Nepal and Philippines to identify the communications needs of indigenous people, while helping empower indigenous populations in media initiatives. This multinational research project used a participatory and inclusive methodology to produce a list of priority recommendations to strengthen the capacity of participants and their communities. Indigenous researchers and indigenous peoples’ organizations took the lead in all five countries and conducted interviews, community consultations and focus group discussions. They focused on a systematic analysis of the contextual issues faced by indigenous peoples, including in terms of representation in state agencies. Using the commonalities of this backdrop, the researchers reported that by the time the assessments were completed and a set of recommendations was completed for each participating country, the indigenous peoples’ groups were able to fully agree on a regional strategy as well.

Infante A: **Citizens and Health Priorities: The Experience in Chile.** In *Participatory Processes for Setting health priorities: 2012; Washington, DC*. Inter-American Development Bank. [ORIGINAL SPANISH]. The Social Preferences study used several techniques and methods to understand the criteria that the public and other stakeholders in Chile used to understand and rank their health problems and needs. All methods were group-based to increase reliability—group priorities are more stable and shared than individual priorities. Qualitative methods were prioritized as it helped avoided biasing respondents with pre-defined categories as quantitative survey tools often do. Methods included town halls, scenario-based focus groups, mail-in surveys, roundtable discussions, opinion polls and panel discussions. A panel of experts was engaged throughout the study to propose items for consideration and questions to ask. The results were combined with the objective cost-effectiveness and disease-burden analyses to update health policy guidelines and establish Chile’s health guarantees.

Head BW: **Australian experience: Civic engagement as symbol and substance.***Public Administration and Development* 2011, **31**(2):102–112. This is a retrospective look at Australian experience in civic engagement. Since the 1980s, most Australian states have been conducting civic engagement or community consultations as a purposeful and planned dimension of policy development. Australian jurisdictions built their system based on normative (rights-based) arguments about civic participation and democratic legitimacy, as well as the programmatic arguments about program effectiveness and improvement. As is often the case for governments that start purposeful community engagement into their policy and programming, there is much progress to be proud about, but important gaps and challenges remain. An important one that many other countries should note is the persisting disenfranchisement of the nation’s indigenous populations. Using four case studies, this paper looks at the development of Australia’s consultative policy processes, the scope and authenticity of various processes and methods and some reasons and mitigation strategies of the disparity of inclusion amongs the indigenous. The author concludes that the Australian example carries examples of building a policy environment that allows effective and efficient partnerships with civil society and build build civic capacity to solve the country’s problems.

Hansson LF, Norheim OF, Ruyter KW: **Equality, explicitness, severity, and rigidity: the Oregon plan evaluated from a Scandinavian perspective.***Journal of Medicine and Philosophy* 1994 19(4):343-366. Oregon proposed a controversial social experiment in which a list of medical conditions-intervention combinations were prioritized and to allow more people to join Medicaid. The lowest 17% of the prioritized list was not reimbursed for. The community was asked to provide input at town meetings, and this was integrated with public desirability ratings of health states, medical judgment of treatment efficacy, and discretions of the Health Services Commissioners. The author looks at Oregon’s plan and evaluates it according to Norway’s more egalitarian health care model. While the explicitness in the Oregon’s prioritization process is a definite strength, Norway incorporates more subjective metrics of disease severity. In the conclusion, the author argues that the Oregon plan’s rigidity can lead to unfair treatment at the individual level and offers a selection rule to address that problem.

Government of Chhattisgarh: **Human Development Report: Chhattisgarh. New Concept Information Systems. 2005.** New Delhi, India. The 2005 human development report from Chhattisgarh, one of the newest states of India describes the process by which the state defined the mandate of their government agencies. This process included broad stakeholder participation and extensive consultations in hundreds of village on a comprehensive set of areas, including health. Participation was created in every step of the way, from conceptualization and guidance to training and report writing. Consultations occurred at the village level, using maximally inclusive consultation formats. Each village had three focus groups: a general group, a marginalized group and a highly marginalized group. Village residents were trained as facilitators to collect, compile and report on data in their regions and had to have at least one facilitator who was a member of the severely marginalized Scheduled Castes and Tribes. The priorities that were eventually defined were assessed and weighed inclusively with marginalized populations at district level consultations. Although a healthy female representation was achieved at the focus groups, practically no consultations talked about women’s health issues. In fact, women’s health and mental health issues were added in by the team compiling the health report at the state level.

Williams JJ: **Citizenship, community participation and social change: The case of area coordinating teams in Cape Town, South Africa.***IDS Bulletin-Institute of Development Studies* 2004, 35(2):19. This is a municipal level example of creating policy changes though consultative processes, and as above, and as above, we see positive value as well as challenges. Williams described and evaluated Areas Coordinating Teams (ACTs), teams that act as mediums to represent pubic voices in local governance matters in post-apartheid Cape Town. ACTs were established to empower historically marginalised and excluded communities by inviting them to meet a broad representation of the city officials and raise demands, issues and complaints to them. However, ACTs appeared to be functionally truncated, institutionally manipulated and structurally limiting and merely served to ratify rather than influence official behaviour. The author describes that the discussions at these ACTs are completely non-binding, the officials were not obligated to attend, and there were no mechanisms to hold the Council accountable to decisions achieved at the ACT meetings and implement community-driven policy change. He concluded that the ACTs have remained largely a political idea and, structurally have not yet become part of the City’s mode of management and have remained only symbolic vestiges of community involvement. For ACTs to become effective instruments of fundamental social change, the city government needed to support ACTs by making them binding and compelling officials and councillors to attend and take seriously scheduled meetings and related development planning initiatives.

This section described some recent examples of a participatory process of policy building. Several studies had a retrospective look at efforts of inclusion that their jurisdictions had created. The common theme in the literature on these studies was that, when the communities under study participated in running the research project, the policies created were inclusionary. Both papers from a multinational or international perspective showed this. Conversely, as long as engagement and inclusion remained the mandate of the government solely, participation became hollow or incomplete, such as in the Australian states and in Cape Town, South Africa.

#### Discussion

A few of the studies included above undertook a one-time participation research project to feed into their policy and provided a detailed description of their efforts of making inclusion. The Indian state of Chhattisgarh used extensive consultations to create what may have been the government’s first mandate. A critical eye on the results will uncover an interesting point. The report noted that mental health and women’s health did not come up in the discussion, and were added on at the consolidation phase. This brings up two issues. Firstly, we know from Welbourn’s (1992) paper that women’s issue will probably drown in a cacophony of men’s issues or family issues. It seems that the consultations did not make the space for women to feel internally included. Secondly, another more objective source of priority data should be used to complement the priority information from the consultations (see Further reading). A *post priori* modification may make the results more complete, but they risk the legitimacy of the final product by putting a researcher’s modification at par with extensive community consultations. An objective method of making modifications should be created before the consultations began. Infante’s (2012) work on Chile’s health system policy had extensive consultations carried out by the government for the expressed aim to change policy. They had multiple instruments that they utilized. However, there was little evidence that the Chilean government prioritized marginalized groups. Finally, Oregon conducted a rationing exercise using citizen’s feedback. This was to establish priorities among pre-determined services instead of determining what the priority needs are. Hansson (1994) compares two ways of doing this kind of prioritization, each one holding up a different set of values (see Underlying values and frameworks for participatory priority research above).

### Challenges and reflection on inclusive policy making

Next, we explore the challenges and reflections on issues that researchers have encountered in participatory consultations and inclusive policy making. The challenges considered in the literature can be classified under three themes, which may be mapped to the stage before, during, and after the consultations, in that order. The first theme revisits the issues of recruitment ahead of the consultations, specifically the challenge of deciding how broad participation should be. It looks at what groups, individuals and issues should be included and whether having more is necessarily better. The second theme brings us back to the instruments used during the consultations. The literature suggests that choosing an instrument is not as simple as picking it from a list, rather populations need to be engaged in a broader sense of the word. On their own, instruments are vulnerable to manipulation (Peterson 2011, above) and we now see that even effective instruments can create varying degrees of inclusion depending on other factors. Yet, allowing the instruments and processes to be exploited may actually be the very point of participation, provided that everyone gets to do this equitably. The final theme looks at issues after consultations have been completed. The first issue builds on the discussion in From participatory consultations to inclusive policy about translating consultation results for policy. We look at the theoretical challenge of making sure every person or group’s input is faithfully communicated to policymakers. We also look at evaluations of and reflections on whether the participation processes sufficiently achieved the goals of inclusion. The final paper annotated looks at the effect of consultations on the participants.

#### Who to consult and other issues before consultations

Macpherson CC: **To strengthen consensus, consult the stakeholders.***Bioethics* 2004, 18(3):283–92. When CIOMS, an international non-governmental agency established by WHO and UNESCO, revised their guidelines for biomedical research, they did it without consulting any stakeholders. Macpherson realizes that the exclusion may be unintentional (logistical difficulties or political failures), but also suggests that it may be because of the relativist-universalist debate: how to invite comments from all interested parties and then build consensus among them without stifling someone’s opinions? She simultaneously dilutes the prospect of universalism as well as the concern of relativist slippery slope idea by noting that consensus building is not achieving unanimous assent but rather a stepwise, dynamic process that is meant to educate and inform through public deliberations towards creating bridges of understanding. She concludes that participatory methods that facilitate capacity building can create these bridges and can help generate broad consensus. Organizations such as CIOMS that aim to represent others in society have an obligation of consensus building.

Adato M, Hoddinott J, and Haddad L: **Power, politics, and performance: Community participation in South African public works programs***,* v*ol. 143.* Washington DC: International Food Policy Research Institute 2005. Drawing on data from 101 public works projects and 8 in-depth case studies in South Africa, the authors show that, despite people accepting the importance of community participation, not everyone believes it is appropriate. They observed that community members often did not have the necessary skills or training, and as such were frequently given only community-worker liaison roles. They also noted that project managers excluded community members from management tasks because the managers did not think they understood efficiency or the overall project objectives. However, the authors shared an important empirical finding: even *de facto* participation had some statistically significant benefits for the community provided that management maintain regular communication with communities. The authors propose to either improve the participation process or just reduce the community’s role to liaison roles to get full use of the benefit without promising more than they want to deliver.

Fine JD, Owen D: **Technocracy and democracy: Conflicts between models and participation in environmental law and planning**. *Hastings Law Journal* 2005, **56**(5):901. Lay persons may not appreciate (and may be apathetic about) the risks and factors involved in certain decisions, so their views might be at odds with legal-, risk- or science-based decisions made by officials. This lack of understanding is even more pronounced for disadvantaged communities who may live in areas with greater environmental risks, but have lesser time or understanding to engage in the decision making process. Instead of using this as a reason for not involving the public, the authors argue that this should prompt policymakers to avoid the overly technical ways of making policy and engage the community in broad outreach and education. This will results in good policies and a more informed public.

#### When inclusive instruments can be excluding: challenges during consultation

Peterson ND, Broad K, Orlove B, et al.: **Participatory processes and climate forecast use: Socio-cultural context, discussion and consensus.***Climate And Development* 2010, 2(1):14–29**.** This study offers a meta-analysis of participation, by looking at the interaction between participation and the socio-cultural environment around participatory research using two case studies from Brazil and Uganda. The authors describe the pull and push factors for participation. These are (1) the diversity of goals and outcomes that motivate participation, including desire for consensus, social networking and community building, and (2) the social norms of interactions that hinder participation (such as alliances, pre-meetings, language). Facilitators that have idealistic goals for a perfectly equitable discussion are often surprised by these socio-cultural barriers. However, the authors say that the barriers are an unavoidable characteristic of participation, and that they are necessary since they motivate participation and are rewarding for participants.

Williams M: **Discursive democracy and new labour: Five ways in which decision-makers manage citizen agendas in public participation initiatives**. *Sociological Research Online* 2004, 9(3). This paper presents the counterexample of Peterson (2010) where the authorities do not merely acts as facilitators of the discussion but are actively participating to affect the discourse and the results. Williams observed how a the authorities running a local initiative attempted to manage differing agenda and to bridge tensions between their own ideas, opinions and values and those expressed by the participating public in order to try and achieve consensus on their own terms. The specific methods he observed included decision makers pledging to address a certain issue; switching the force of the participants’ agenda towards the agency’s objective; pleading ignorance; and attacking. Williams characterized the process as “discursive democracy”: citizens may dialogue, but only the elected representative retains policy formulation rights.

Ndiaye P, Ndiaye NM, Diongue M, et al.: **Community participation for a latrine project in Senegalese rural area**. *Sante Publique* 2010, 22(1):147–154. This research analyzed the participatory process and showed the importance of consultation during the project implementation phase of a project. It studied community participation around a 3-year failed project through a descriptive and analytical survey of the project and individual and group interviews. The authors found that community participation to make decisions about which activities would be undertaken did exist. However, consultation was limited to areas of needs assessment, mobilization and management of resources, as well as monitoring and evaluation but without a high level of participation in the implementation phase. The authors noted that projects often recruit community participants but then have serious shortcomings in participation at subsequent phases, especially during that of implementation. Moreover, they showed that generic participation strategies did not support the inclusion of poor and disadvantaged populations, a majority of whom have low health education and literacy. The authors concluded that health professionals need to organize community representatives and train them to be empowered partners in their own projects. This would make them feel that they have stakes at all phases of the project.

Pyett P: **Working together to reduce health inequalities: reflections on a collaborative participatory approach to health research.***Australian and New Zealand Journal of Public Health* 2002, 26(4): 332–6. This paper discusses participatory approaches to health research, outlines key collaborative processes on a continuum from advocacy to action research. The author identifies methodological tensions (e.g., representation, disagreements) and ethical issues (e.g., non-maleficence, informed consent) that arise when using such approaches. The discussion focuses on marginalized and indigenous populations and makes a strong argument for inclusion by disadvantaged and marginalized groups because of the effects of social inequalities on health.

#### Post-consultation: reflections, challenges and evaluations

Strobl J, Bruce N: **Achieving wider participation in strategic health planning: experience from the consultation phase of Liverpool’s ‘City Health Plan’**. *Health Promotion International* 2000, 15(3):215–25.

Rasanathan K, Posayanonda T, Birmingham M, Tangcharoensathien V: **Innovation and participation for healthy public policy: the first National Health Assembly in Thailand.***Health Expect* 2012, 15(1):87–96.

Mubyazi GM, Mushi A, Kamugisha M, et al.: **Community views on health sector reform and their participation in health priority setting: case of Lushoto and Muheza districts, Tanzania.***Journal Of Public Health* 2007, 29(2):147–156.

These three studies focus on the district or municipal level of government policy. Each of these asked consultation participants to evaluate the success of consultative health policy planning forums led by their governments. Liverpool’s City Health Plan undertook consultations to create wider participation. The first paper used questionnaires to ask participants to assess the success of that consultation process in achieving its goals, allowing participants to define their own indicators. While the consultations were widely appreciated, many participants asked for more opportunity to understand the implications of the plan itself. In Thailand, the government created a National Health Assembly (NHA) as an innovative, participatory forum for making health policy with multiple stakeholders, including civil society. Using their own experience and document analysis, the authors state that the NHA successfully brought together various groups including groups often marginalized in policy making but significant challenges remained in ensuring full participation of interested groups and in implementing, and monitoring the impact of, the resolutions passed. The Tanzania study conducted household level group discussions to collect community views on health sector reforms (HSR) and priority setting in Tanzania. They used a sound sampling strategy that included various villages, wards and development committee members. The HSR did not meet several community needs but its development committees were also seen to function poorly as compared to other local community participatory priority-setting structures. More effort was needed to enhance community knowledge, trust and participation in the health sector programmes at all levels. These three papers show that one of the best way to evaluate whether consultations were inclusive is to ask the participants themselves. The feedback can be used to conduct stronger consultations in the future. However, it should be noted that the Tanzania study was more participatory than the Liverpool plan since they sought feedback from a representative sample instead of going back to those that had already been consulted and might be more favourable.

Stronks K, Strijbis AM, Wendte JF, Gunning-Schepers LJ: **Who should decide? Qualitative analysis of panel data from public, patients, healthcare professionals, and insurers on priorities in health care.***BMJ* 1997, 315(7100):92–96. This paper looks at the multiplicity of views and opinions policymakers are faced with after stakeholder consultations, and the problems with synthesising and consolidating them for a policy goal. The authors organized a series of panels for the stakeholders and asked them to ration 10 services under a limited budget, and analysed the results qualitatively. Healthcare professionals agreed on the importance of the services, but differed on who will pay for them. The patients economised by limiting universal access to preventive and acute services. The “public” panels instead excluded cheaper services, emphasising that health behaviour is an individual’s responsibility. The authors noted that the main difference between the stakeholders seems to be the extent to which the parties took the principle of equal access into consideration. They concluded that including all stakeholders does not necessarily lead to more equitable or broadly supported outcomes.

O’Keefe E, Hogg C: **Public participation and marginalized groups: The community development model.***Health Expectations* 1999, 2(4):245–254. This study looked at the difficulty of consolidating between health needs of individuals and public health needs of groups, as well as the priorities of different groups of people. It presented the experience of HealthLINK, a community health council-based project that allowed house-bound older people to share their views in planning health and social care. Health officials managed to access the views of a highly excluded group of people, but their approach also brought up many conceptual tensions. For instance, the project was based in helping many individuals and as a result became about health services related needs and not underlying non-health issues. Moreover, the researchers noted that discovering clients’ health needs now usually have to be followed by rationing and prioritization. Problems arise because “need” and priorities are defined differently by healthcare workers and patients. These are not only moral differences but also bring up issues of unjustifiable inequalities. They suggest that building consensus on what is a priority needs becomes an important part of addressing needs. Community participation here can be especially empowering (see Macpherson 2004, above).

Attree P, French B, Milton B, Povall S, Whitehead M, Popay J: **The experience of community engage-ment for individuals: a rapid review of evidence.***Health Soc Care Community* 2011, 19(3):250–60. Despite how widespread community engagement approaches and their reviews have become, relatively few attempts have reviewed the evidence on the impact that participation has on the lives of individuals involved. This paper provided a unique perspective on participation by analyzed 22 studies containing empirical data on the subjective experience of consultation participants at consultations that were meant for addressing social determinants of health. The findings suggested that the majority of participants perceived benefits in their physical and psychological health, self-esteem and feeling of empowerment. However, it also suggests that there are several unintended negative effects of engagement for some individuals that may risk their well-being. Other than consultation fatigue and disappointment, participants complained of exhaustion, stress and energy levels, as well as of material resources such as time and money. This was especially noted by individuals with disabilities. At a personal level, individuals balance the benefits against the harms. The paper was a useful reminder that there are negative effects of participation and strengthen the call for self-reflection.

#### Discussion

This section has presented a reflexive look on most of the aspects of inclusive consultation and policymaking research. As earlier, the recruitment and equal participation continued to be some of the most important themes. It is important to note that the first few articles are not just reflective pieces but bring up some very important challenges with creating participatory consultations and inclusive policies. Macpherson (2004) talks about relativism of priorities, Adato et al. (2005) about the lack of vision and knowledge in the community, and Fine & Owen (2005) talk about the apathy and disconnect in the general population. These are some of the most common reasons that governments and policymakers appeal to, whether consciously or implicitly, when they restrict the input of communities in their policies. However, we see from the same papers that these are not reasons to decrease participatory practices but to increase participation (Adato et al. 2005) or, better yet, increase the capacity and education of a community for them to realized their stakes and participate on their own terms. Further, these papers also suggest that participation is not about the number of people that provide input or just replicating instruments that other researchers may have found helpful. These are necessary but not sufficient factors for creating inclusive policies. If a jurisdiction has the sincere will to give the decision making power to the marginalized, they need to let go of their own policy goals and predictability of the results, open themselves up for critique and criticism and inculcate in the community the confidence that their input will be respected.

The next few papers bring up the issue of equal participation when reflecting on the consultations themselves. It is important to note the several respects in which the papers compiled in this section extend of that idea. Participation has to be from the earliest conceptualization phase of a project to the evaluation after the project is completed, as well as all the steps in between. It should allow for expressions of socio-cultural dynamics but should make sure that these dynamics are not persisting social inequalities, whether they are between two community members or between the researchers and the community.

Some final reflections reaffirm the central role the community—both the persons and the groups—play in a system that promotes inclusion. Although a major tension still exist here (consolidation of views, which we speak to in the next paragraph), we can say with a reasonable degree of confidence that failing to complete an inclusive evaluation after a consultation may risk hurting the credibility of the entire process, even if there was participation at every other phase. An important way of doing this in a transparent way is to engage in an evaluation of their process, see how included participants feel, and how inclusive the policies are. However, Adato et al. (2005) remind us that one challenge will still remains. Community members that do not have the skills or training to understand the substance of their decisions may sometimes want to have a bigger role in making those decision. For this reason, we should take heed of the calls for building capacity in the participants to have reasoned opinions and the confidence to express them when asked.

The issue of consolidation of issues brought up in consultations still remains. . Whereas the question of recruitment deals with the *size* of the universe of needs, the consultations may result in an infinite number of needs, desires and priorities that have to be faithfully reflect on the policy. This knowledge translation may require prioritizing and omitting *within* the universe, sometimes between contradicting views. The consolidation of a multitude of views into one report or policy represents a phase that is highly susceptible to the researchers’ own biases, or at the very least, their discretion. One of the most important ways to ensure that the needs and priorities of the community are not lost at this level is to go back to the community a second time to validate the results and findings of the original consultations.

### Further reading

Several of the studies presented above recommended using multiple methods and sources of evidence. This final section suggests a few additional sources of data that can be used to increase the validity of the results of community participation. These evidence sources can also help with the final stage of rationalizing and compiling priority data for use by policy makers. Policy makers who discredit the strength of consultative data might be more willing to accept it if the finding from the two sources match. Researchers can use instances where they do not match to explain the paradox to the policymakers. We saw such paradoxes appear in the examples of what were otherwise very consultative processes, such as when the Government of Chhattisgarh (2005) missed out women’s health and mental health issues completely, or when Oregon refused covering for conditions that seemed of a lower priority when it may just be that it is a rare but important disease. The alternative sources of data are briefly described below with a few references. Making a definite case for or against any one of them will require a systematic literature review, and is beyond the goals of this bibliography. As such, there is a brief description provided and some references to help the reader begin to understand the potential options available.

1. A systematic literature review of past research on community needs in healthcare can develop a strong evidence base for some common issues across the board. It can also be used to combine the results of various types of data and analyse it statistically.
  Rees R, Harden A, Thomas J, Oliver S, Kavanagh J, Burchett H: HIV health promotion and men who have sex with men (MSM): a systematic review integrating ‘qualitative’ studies and trials [abstract]. 12th Cochrane Colloquium: Bridging the Gaps; Ottawa, Canada: The Cochrane Collaboration; 2004; Oct 2–6
  Dowrick C, Gask L, Edwards S, Aseem S, Bower P, Burroughs H, et al.: Researching the mental health needs of hard-to-reach groups: managing multiple sources of evidence. BMC Health Services Research 2009, 9:226
2. Children generally are not considered capable of giving consent, so consulting them has to be done differently. Many measures to involve children are based on giving them respect and listening to them.
  Cavet J, Sloper P: The participation of children and young people in decisions about UK service development. Child: Care, Health and Development, 2004, 30(6): 613–621
3. Ethnographic Studies can be used in general consultations, but may be more important to used to get a better idea of the needs of intellectually disabled individuals who do not have the capacity to make high level decisions. The paper suggests using photographic images as an alternative methods to understand the experience of these individuals.
  Ottmann G, Crosbie J: Mixed method approaches in open-ended, qualitative, exploratory research involving people with intellectual disabilities: a comparative methods study. Journal of intellectual disabilities 2013, 17(3): 182–97
4. Essential health packages can be based on several different reasons, but generally respond to many urgent and acute needs of a population. A survey of these conducted in China pointed to some patterns around the world.
  Yang, Li et al. 2009a. Strategies to Develop an Essential Healthcare Package: Background, Strategy and Effect. Evidence-Based Medicine in China, 9(6): 599–609 [ORIGINAL MANDARIN]
  Yang, Li et al. 2009b. Strategies to Develop an Essential Healthcare Package: Definition, Package and Criteria. Evidence-Based Medicine in China, 9(6): 599–609 [ORIGINAL MANDARIN]
5. Rapid needs assessment have been used in disaster-stricken areas for over a decade now and can generate some valid results.
  Springgate, BF, Allen C, Jones C, et al.: Rapid Community Participatory Assessment of Health Care in Post-Storm New Orleans. American Journal of Preventive Medicine 2009, 37(6):S237-S243
6. Many consultations invite representatives of CSOs or NGOs to consultations instead of clients of those CSOs. This has advantages and disadvantages, but can certainly be an easier way of understanding the needs and priorities of a specific population.
  De, R. (2006). The impact of Indian e-government initiatives: Issues of poverty and vulnerability reduction. Regional Development Dialogue, 27(2), 88–100.

### Conclusion

This bibliography adds value to the research in two aspects. Firstly, it brings in research on participatory consultations and applies it to policy-making or priority-setting processes, within the context of the marginalized. This is becoming increasingly important because, (a) participatory practices are seen as providing better outcomes and more sustainable projects, and (b) since development aid is declining while disparities are only becoming more pronounced, many more nations are looking at serving the marginalized both as a moral obligation and as a political priority. Second, in covering fundamental conceptual framing and instruments to case studies and evaluation techniques, this paper has synthesized an extensive and varied topic of research into a structured, practical tool that governments and NGOs can apply when they are initiating a participatory policy-making program.

All inclusive methods should start with a clear goal and a framework that shows how the goal will be achieved. The literature delineates between philosophical foundations based on value, conceptual frameworks that tie various variables to each other, justifications for prioritizing the marginalized, all within the context of international development. Some references that are provided demonstrate how institutions and nations have codified the attainment of certain goals and minimum requirements on how it will be done.

The bibliography then surveys the instruments that can be used to create inclusion. The cited studies include papers that talk about how to recruit participant in an unbiased fashion, how to consult openly and legitimately, and how to transmit the results to policy-makers reliably. We learn that inclusion is not a series of methods, but a sustained partnership. All the instruments that were used to recruit, consult and present to policy-makers had their own set of benefits and drawbacks, but choosing the input of the community on what kind of instrument they would like to use would create better inclusion. Several case studies at the municipal, state, national and international levels are provided to help the reader learn from the parallels and of the variations. These case studies shared the benefits of using participatory processes and the risks of not. We look at several more evaluative studies that, taken together, can help us critically reflect on each stage of the process. Consulting offers several benefits but also carries theoretical and operational challenges. The task of the researcher is to find the group of methods that can maximize the benefits and minimize the challenges. The challenge for policy-makers is to get the kind of civic engagement to make it a worthwhile endeavor.

## Abbreviations

UN: United Nations
NGO: Non-governmental Organizations
PRA: Participatory rural appraisal
SES: Socioeconomic status
CVD: Cardiovascular disease
NYUHHC: New York University Health and Hospitals Corporation
HIV: Human Immunodeficiency virus
ACT: Areas Coordinating Teams
CIOMS: Council for International Organizations of Medical Sciences
WHO: World Health Organization
UNESCO: United Nations Organization for Education, Science and Culture
NHA: National Health Assembly
